# Mouse adipose tissue stromal cells give rise to skeletal and cardiomyogenic cell sub-populations

**DOI:** 10.3389/fcell.2014.00042

**Published:** 2014-08-25

**Authors:** Cécile Dromard, Corinne Barreau, Mireille André, Sandra Berger-Müller, Louis Casteilla, Valerie Planat-Benard

**Affiliations:** ^1^Centre National de la Recherche Scientifique (CNRS) UMR 5273, STROMALabToulouse, France; ^2^Université Paul Sabatier de ToulouseToulouse, France; ^3^Institut National de la Santé et de la Recherche Médical (INSERM) U1031, STROMALabToulouse, France; ^4^Etablissement Français du Sang (EFS) Pyrénées-Méditerranée, STROMALabToulouse, France

**Keywords:** Adipose tissue stem cells, cardiac and skeletal myogenesis, *in vitro* differentiation, cell plasticity

## Abstract

We previously reported that adipose tissue could generate cardiomyocyte-like cells from crude stromal vascular fraction (SVF) *in vitro* that improved cardiac function in a myocardial infarction context. However, it is not clear whether these adipose-derived cardiomyogenic cells (AD-CMG) constitute a homogenous population and if AD-CMG progenitors could be isolated as a pure population from the SVF of adipose tissue. This study aims to characterize the different cell types that constitute myogenic clusters and identify the earliest AD-CMG progenitors *in vitro* for establishing a complete phenotype and use it to sort AD-CMG progenitors from crude SVF. Here, we report cell heterogeneity among adipose-derived clusters during their course of maturation and highlighted sub-populations that exhibit original mixed cardiac/skeletal muscle phenotypes with a progressive loss of cardiac phenotype with time in liquid culture conditions. Moreover, we completed the phenotype of AD-CMG progenitors but we failed to sort them from the SVF. We demonstrated that micro-environment is required for the maturation of myogenic phenotype by co-culture experiments. These findings bring complementary data on AD-CMG and suggest that their emergence results from *in vitro* events.

## Introduction

The inexorability of heart failure has prompted the development of cell therapy as a new strategy to replenish the pool of dead cardiomyocytes. A prerequisite in such efforts is to determine which progenitor cells can be differentiated into a functional cardiac phenotype. Resident cardiac stem and progenitor cells have been identified in adult mammalian myocardium (Askari et al., 2003; Beltrami et al., 2003; Martin et al., 2004; Matsuura et al., 2004; Messina et al., 2004; Laugwitz et al., 2005) and a phase 1 clinical trial showed that autologous cardiac stem cells may be efficient in patients with ischemic cardiomyopathy (Bolli et al., 2011). However, their use requires a previous isolation step for *in vitro* expansion, which is invasive for the heart to be treated.

Currently, a variety of other autologous adult progenitor cells that could generate differentiated cells beyond their own tissue boundaries are of great interest. Skeletal muscle myoblasts and bone marrow-derived cell subsets (hematopoietic stem cells, mesenchymal stem cells) were tested as potential sources of cardiac progenitors for cell replacement therapy and yielded positive results in infarcted myocardium of various animal models. However, despite integration and survival their predominant *in vivo* effect may be related to neoangiogenesis or supportive effect (for review, see Menasche, 2011). Indeed, with the exception of Spoc cells (Skeletal-based precursors of cardiomyocytes) isolated on the basis of several surface markers from adult mice skeletal muscles, that have demonstrated their potential to differentiate into beating and functional cardiomyocytes both *in vitro* and *in vivo* (Winitsky et al., 2005), it is now recognized that both skeletal myoblasts and bone marrow cells lack the degree of plasticity allowing them to widely convert into cardiomyocytes *in vivo* (Reinecke et al., 2002; Scherschel et al., 2008).

Previous studies highlighted the existence of adipose tissue derived progenitor cells possessing cardiogenic potential and being able to promote myocardial regeneration (Planat-Benard et al., 2004; Yamada et al., 2006; Leobon et al., 2009). Indeed, clusters of myogenic cells spontaneously emerge from culture of the crude stromal vascular fraction (SVF) of adipose tissue in semi-solid medium. The clusters contain cells that exhibit pace-maker contractile activity, are responsive to chronotropic agents and express different cardiac markers such as transcription factors and specific contractile proteins (Planat-Benard et al., 2004). Until now, the origin of adipose derived-cardiomyogenic cells (AD-CMG) has not been clearly determined. Indeed, we and other teams have tried to identify in adipose tissue, progenitor cells owning the potential of cardiomyogenic differentiation, but only partial phenotypes were established from cells freshly prepared from SVF and the progenitors of AD-CMG have still never been identified *in situ* (Yamada et al., 2006, 2007).

The present works aims to identify and characterize the earliest AD-CMG progenitors in myogenic clusters *in vitro* for subsequently use these hallmarks in order to prospectively isolate such progenitors from SVF of adipose tissue.

## Materials and methods

### Ethical approval

Eight-week-old male C57Bl6J mice (Harlan) were housed in a controlled environment (12-h light/dark cycle at 21°C) with free access to water and a standard chow diet (UAR). All procedures were performed in accordance with the European Community guidelines for the care and use of laboratory animals (EEC/No. 07430).

### Isolation and culture of adipose derived cells

Mice were euthanized by cervical dislocation. Brown interscapular and white peritoneal or inguinal adipose tissues were withdrawn and subjected to mechanical dissociation and digestion in DMEM-F12 medium (Invitrogen, Carlsbad, USA) supplemented with bovine serum albumin (BSA) (2%) and 2 mg/ml collagenase A (Roche Diagnostics), for 30 min at 37°C. After elimination of undigested fragments by filtration through 25-μm filters, cell suspension was centrifuged at 486 g for 10 min to separate floating mature adipocytes from the SVF. SVF was incubated in erythrocytes lysis buffer (ammonium chloride solution) (StemCell Technologies) for 5 min at 4°C and washed in PBS. SVF cells were counted and used for further analysis (flow cytometry) or cultured in semi-solid medium as previously described (Leobon et al., 2009). Briefly, freshly prepared SVF cells were plated (27000 cells/mL) and maintained for 2 weeks in methylcellulose (Methocult M3534, StemCell Technologies), corresponding to semi-solid culture condition.

For some experiments, myogenic clusters were transferred from semi-solid to liquid culture condition (Leobon et al., 2009). Indeed myogenic clusters of cells were dissected under an inverted phase-contrast microscope and were plated (1500 cells/cm^2^) into 30-mm culture dishes coated with 0.1% gelatin and cultured in BHK21 medium containing fetal bovine serum (10%), supplemented with b-mercaptoethanol (1024 M), glutamine (2 mM), pyruvate (1 mM), non-essential amino acid (0.1 mM), and a solution of amphotericin (0.25 mg/mL), penicillin G (100 U/mL), and streptomycin (100 mg/mL), corresponding to liquid culture condition. In that case passaging was performed by collecting non adherent cells from the culture medium that were successively plated to reach passage 15 (P15). For flow cytometry, myogenic clusters were picked from methylcellulose under an inverted phase-contrast microscope, washed with PBS and mechanically dissociated.

### Cell phenotyping

Freshly-isolated SVF cells or AD-CMG were stained in PBS supplemented with 0.5% new calf serum and FcR Block reagent (StemCell Technologies). Sextuple stainings were performed by direct immunofluorescence with conjugated rat anti-mouse monoclonal antibodies (listed in Table 1) as compared with their matched immunoglobulin isotype controls (BD Biosciences). Cells were washed in PBS and analyzed on a fluorescence-activated cell sorter (FACS Canto II, Becton Dickinson). Data acquisition and analysis were performed using FACS Diva software (Becton Dickinson).

**Table 1:** **List of conjugated monoclonal antibodies used for cell phenotyping**.

**Antibodies**	**Clone**	**Conjugates**
CD29^a^	Ha2/5	FITC
CD31^a^	MEC13.3	APC
CD34^a^	RAM34	A647
CD44^a^	IM7	PerCp-Cy5.5
CD45^a^	30-F11	APC-Cy7
CD73^a^	TY/23	PE
CD81^a^	Eat2	PE
CD90^a^	53-2.1	FITC
CD117^a^	2B8	PerCp-Cy5.5
CD133^b^	13A4	APC
CMH1^a^	AF6-88.5	PE
CMH2^a^	AF6-120.1	FITC
Flk1^a^	AVAS 12α1	PerCp-Cy5.5
Sca1^b^	D7	PE-Cy7

a *Antibodies obtained from BD Biosciences, San Jose, CA*.

b *Antibodies obtained from eBiosciences, San Diego, CA*.

*FITC, fluorescein isothiocyanate; PE, phycoerythrin; PerCP, peridinin chlorophyll protein; PE-Cy7, phycoerythrin cyanine 7; APC, allophycocyanin; APC-Cy7, allophycocyanin cyanine 7*.

### Immunochemistry

Cultured cells were washed out from methylcellulose with PBS then fixed with paraformaldehyde (3,7%). Cells were stained with antibodies against cardiac troponin T (c-TnT clone 13-11), myogenin (Santa Cruz, clone M-225), and Numb (Abcam) as compared with their controls (matched immunoglobulin isotype for monoclonal antibody and primary antibody omission for polyclonal antibodies). After washing, conjugated secondary antibodies were added for 1 h. Nuclei were stained with DAPI (1/10,000). Cell staining was observed with a fluorescence microscope (DMRB Leica) and analyzed with the image analyzer software NIS (Nikon).

### Western blot analysis

Extensor digitorum longus muscles and hearts were dissected from C57Bl6J mice, minced with scissors, washed in PBS, and ground in RIPA lysis buffer containing protease inhibitors. Samples were incubated on ice for 1 h and vortexed every 20 min. Protein extracts were then sonicated, centrifuged at 15000 g for 15 min, and protein concentration of the supernatant was determined using the BCA assay (Pierce). 25 μg proteins were loaded on a 12% acrylamide gel. Proteins were transferred on a PVDF membrane using a semi-wet transfer system. Membranes were incubated with mouse anti-cTnT antibody (clone 13-11) and with HRP-conjugated anti-mouse antibody. Membranes were then stripped and probed with rabbit anti-β-tubulin antibody (Abcam) and HRP-conjugated anti-rabbit antibody.

### Detection of cell fusion

SVF cells were labeled with PKH26 using the PKH26-Red Fluorescence Cell Linker kit (Sigma–Aldrich) according to the manufacturer's instructions. In parallel, SVF cells were isolated from transgenic GFP-expressing mice [C57Bl/6 TgN(act-EGFP)OsbC15-001-FJ001] kindly provided by Prof. M. Okabe (Okabe et al., 1997). Equal numbers of GFP- and PKH26-labeled cells were mixed and plated in methylcellulose (5000 cells/cm^2^). As controls, GFP- and PKH26-labeled cells plated separately in methylcellulose. After 5 days of culture, cells were distinguished by fluorescence microscopy (green and red fluorescence respectively) directly in methylcellulose.

### Data analysis

Results were expressed as means ± s.e.m. Data from at least three independent preparations of AD-CMG derived from BAT, WAT-ING, WAT-P, or inguinal derived adipose stromal cells (ASC) were statistically processed with the Wilcoxon test using the Prism 4 software (GraphPad, San Diego, CA). Significance was defined as ^*^p ≤ 0.05, ^**^p ≤ 0.01, and ^***^p ≤ 0.001.

## Results

### Morphological heterogeneity in contractile myogenic clusters

**Figure 1:**
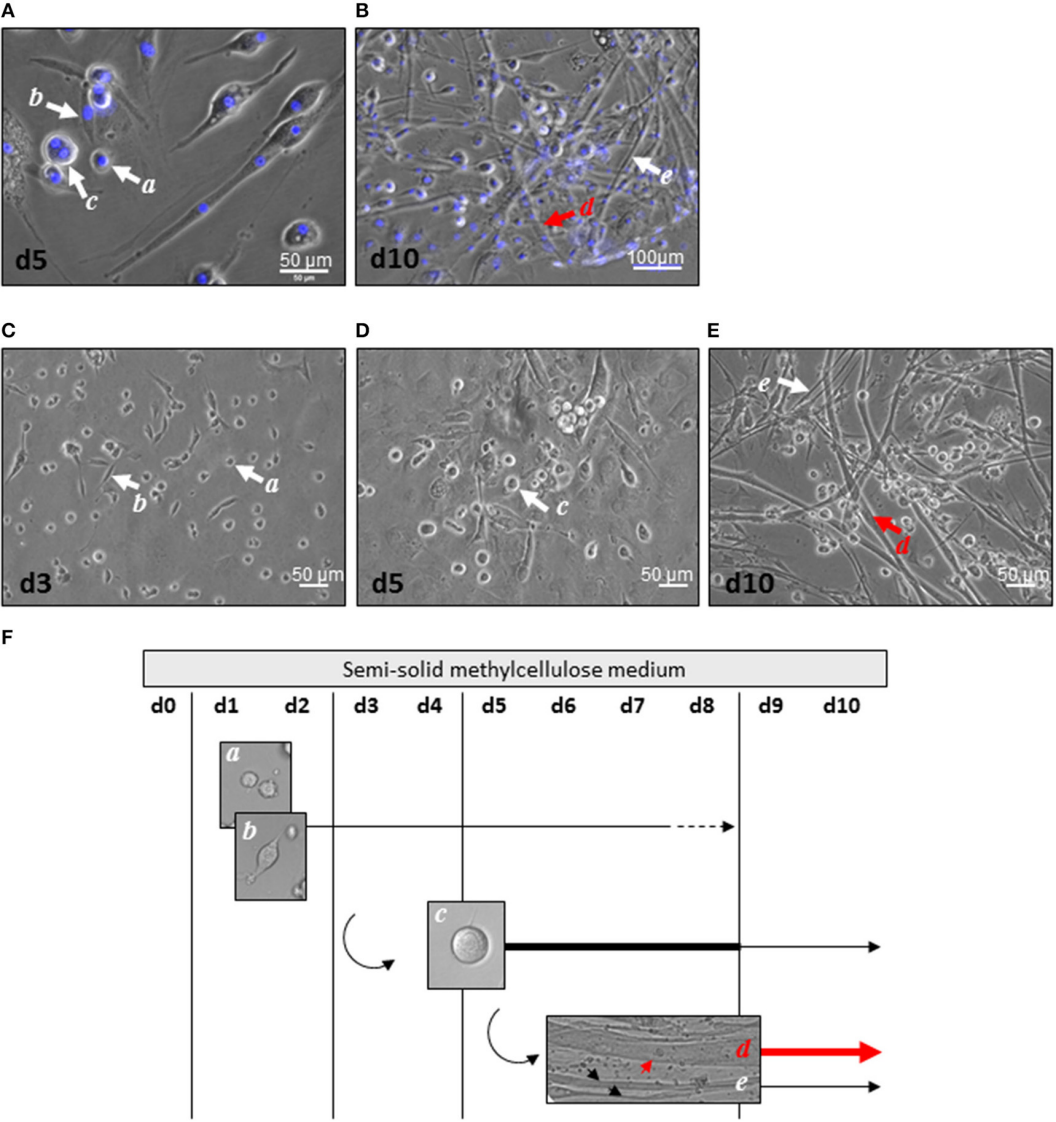
**Cell heterogeneity in myogenic clusters. (A,B)** After 5 days **(A)** and 10 days **(B)** of culture, AD-CMG displayed mono and multi-nucleation associated to various morphologies: small round mono-nucleated cells (*a* cells), bipolar mono-nucleated cells (*b* cells), large round multi-nucleated cells (*c* cells), thick multi-nucleated tube-shaped cells (*d* cells), and thin multi-nucleated tube-shaped cells (*e* cells). Nuclei were stained with Dapi (blue). **(C–E)** Distinct cell morphologies appeared along maturation of myogenic clusters 3 days **(C)**, 5 days **(D)**, and 10 days **(E)** after plating of SVF cells in semi-solid medium (scale bars 50 μm). **(F)** Time course of distinct cell morphologies emergence in myogenic clusters after plating of SVF in semi-solid. Thick lines represent the prevalent cell type, whereas dotted lines represent the minor cell type.

Mascroscopic analysis of contractile myogenic clusters obtained in semi-solid medium revealed cell heterogeneity in size, shape, and nucleus number as initially described (Figures 1A,B). Distinct cell morphologies appeared sequentially, some of them being maintained over time. Small round or bipolar mono-nucleated cells (15 μm diameter) appeared during the first 3 days of culture (Figures 1C,F). From day 5, large round multi-nucleated cells (30 μm diameter) constituted the main cell type in the clusters and some of them displayed spontaneous contractile activity (Figures 1D,F). From day 8, no more mono-nucleated cells could be observed and from day 10, multi-nucleated tube-shaped cells constituted the main cell type in the clusters (Figures 1E,F). Two types of tube-shaped cells could be distinguished; thin elongated multi-nucleated cells, and thick elongated multi-nucleated cells (reaching 17 μm in diameter). The latters displayed spontaneous beating activity, in part in a synchronous way. Our previous data had demonstrated their cardiogenic phenotype sustained by electrophysiological characteristics of cardiomyocytes (Planat-Benard et al., 2004); here, we provided additional functional evidence. Considering that electrical stimuli cannot elicit contractions in cardiomyocytes in the absence of external calcium ions but are capable of doing so in skeletal muscle (Nabauer et al., 1989), we tested the ability of beating cells to develop or not contractions in the absence of extracellular calcium. Replacement of the culture medium for a medium containing the selective calcium scavenger EGTA stopped spontaneous beating and confirmed the dependence of beating cells on extracellular calcium. This effect was reversed by reintroduction of the calcium containing-control culture medium that induced once again spontaneous contractions (Supplemental video online). Thus, spontaneously contracting thick elongated multi-nucleated cells are a population of adipose-derived cells exhibiting a cardiac rather than skeletal-like contractile phenotype.

### Characterization of cluster-initiating sub-populations

In order to evaluate the emergence and persistence of cardiogenic phenotype, expression of cardiac proteins was assessed in each cell type present in the clusters obtained in semi-solid medium or passaged in liquid medium.

Many proteins initially considered as cardiac specific have actually been demonstrated to be expressed in skeletal muscle as well, such as the transcription factor Myocyte Enhancer factor 2C (MEF2C) (Chen et al., 1998; Le Bihan et al., 2006; Potthoff et al., 2007). So, we first verified by western blot the specificity of the tools used as markers of cardiomyogeneity. Among the proteins we tested, the cardiac contractile protein troponin C (cTnT) only, appeared to be exclusively expressed in the mouse heart (Figure S1).

**Figure 2:**
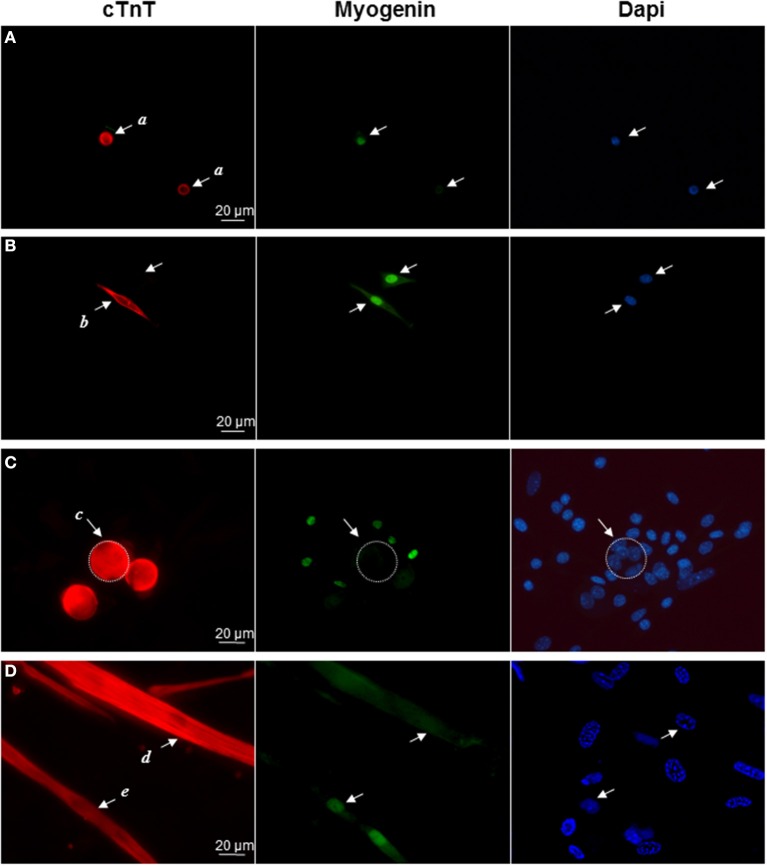
**Differential expression of cardiac and skeletal markers reflect cell heterogeneity in myogenic clusters**. Immunolabeling of cardiac TnC (red) and myogenin (green) in **(A)** small round cells (*a* cells), **(B)** bipolar cells (*b* cells), **(C)** large round cells (*c* cells), **(D)** thick tube-shaped cells (*d* cells), and thin tube-shaped cells (*e* cells). Nuclei were stained with Dapi (blue).

Then we performed double immunocytochemical stainings against the cardiac troponin C and the skeletal transcription factor myogenin, respectively located in cell cytoplasm and nucleus to clearly distinguish specific labeling. In early formed clusters appearing in semi-solid medium, we obtained distinct profiles of expression for these markers associated with distinct cell morphologies (Table 2, Figure 2). cTNT and myogenin were present in cluster-initiating cells, i.e., the small mono-nucleated cells, with various combinations. Some cells appeared exclusively stained with cTNT or with myogenin and somewhat surprisingly, some expressed both markers simultaneously (Figures 2A,B). Whereas thin tube-shaped cells always co-expressed cTNT and myogenin, large multi-nucleated round cells and thick tube-shaped cells exclusively expressed cTNT (Figures 2C,D). Based on these protein expressions and on the functional evidences of their cardiogenic phenotype as described above, we restricted the AD-CMG designation name to the contractile thick tube-shaped cells. Our results suggested that cluster-initiating cells may be intrinsically heterogeneous. During the time course of cluster maturation, it is likely that cTnT(+)myogenin(−) cell populations initially represented in small round cells, then large multi-nucleated cells and later on thick contractile tube-shaped cells (AD-CMG) were cardiomyogenic cells that could be distinguished from cTnT(+/−) but clearly myogenin(+) skeletal muscle cell populations represented in small round-shaped then thin tube-shaped cells. In order to target the progenitor of AD-CMG, we focused on the large round multi-nucleated cell population displaying a cTnT(+)myogenin(−) phenotype that emerged before myotube-shaped cells in semi-solid medium, thus representing a good candidate for AD-CMG progenitors identification.

**Table 2:** **Combinations of cardiac and skeletal myogenic protein expression associated with distinct cell morphologies in cardiomyogenic clusters**.

**Cell phenotype**	**Troponin T**	**Myogenin**
Small round mono-nucleated (a)	+/–	+/–
Small bipolar mono-nucleated (b)	+	+
Large round multi-nucleated (c)	+	–
Thick multi-nucleated tube-shaped contractile (d)	++	–
Thin multi-nucleated tube-shaped (e)	+	+

While emergence of myogenic clusters required a first step of culture in semi-solid medium, for long term expansion myogenic clusters were picked from the semi-solid medium and transferred to liquid medium as reported by Leobon et al. (2009). In liquid medium, differentiated cells adhered to the plastic dishes while large round multi-nucleated cells (the cluster-initiating cells), were non adherent so; subsequent cell passages were performed by harvesting cells in suspension in the liquid medium and replating.

**Figure 3:**
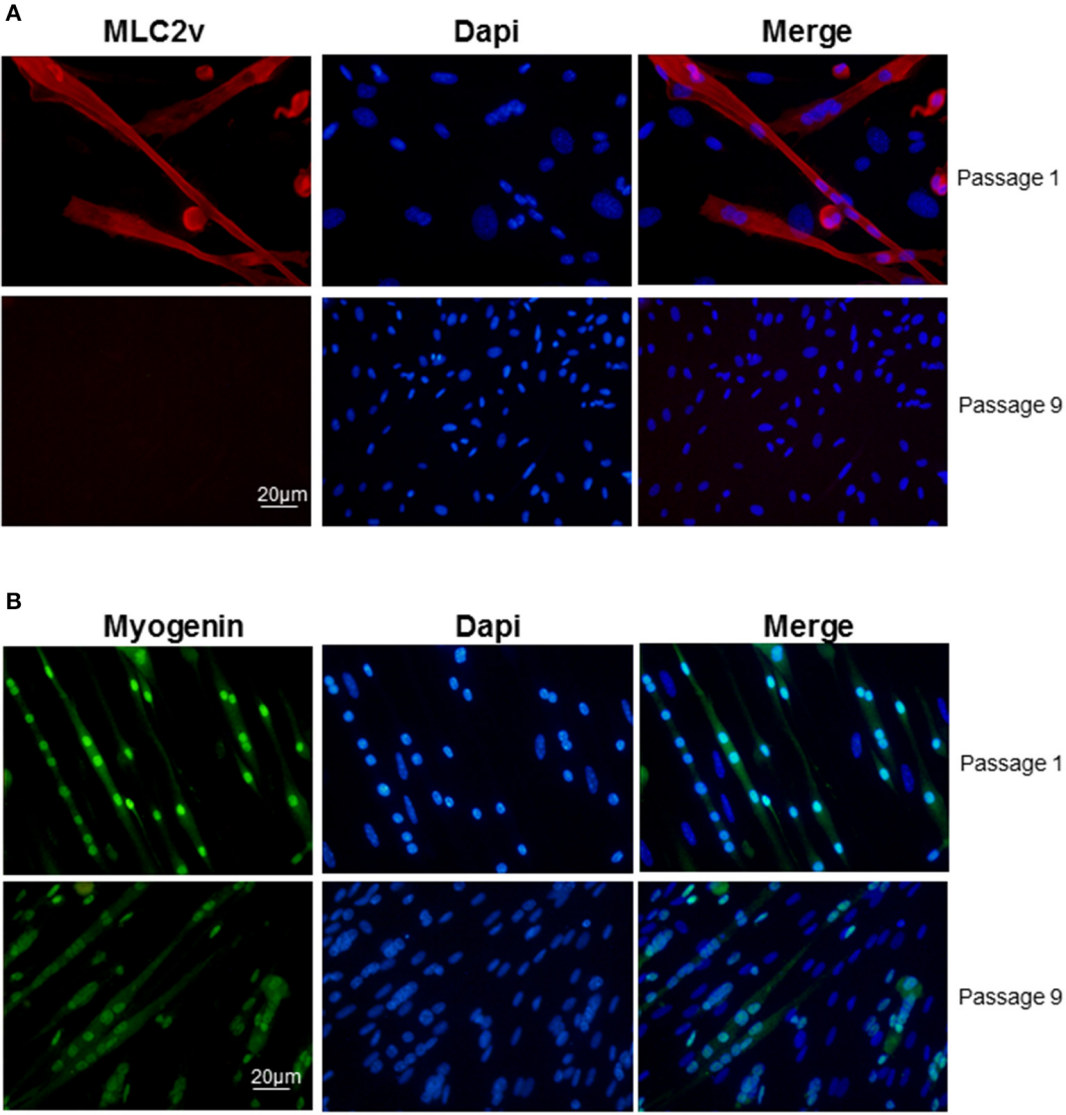
**Decline of cardiac/skeletal myogenic phenotype in liquid culture conditions**. Immunolabeling of **(A)** myogenin (green) and **(B)** MLC2v (red) in myogenic clusters at passages 1 and 9 in liquid medium. Nuclei were stained with Dapi (blue).

Clusters that emerged in liquid medium contained cells that displayed the same morphologies as in semi-solid medium but the cardiac markers cTnT and MLC2v stainings declined progressively and at the end of passage 1 nearly all cells in the clusters expressed myogenin exclusively (Figure 3). Of note, from passage 9, skeletal muscle markers also began to decline (Figure 3B) and after passage 15, skeletal markers could not be detected either. Same results were obtained with the skeletal muscle marker MyoD (data not shown). Taken together, these data pointed out that adipose-derived myogenic cluster displayed important cell heterogeneity and original mixed cardiogenic/skeletal phenotypes during the course of their differentiation. Moreover, it appeared that cardiomyogenic phenotype was transient and progressively declined in favor to skeletal-like phenotype upon large expansion in liquid medium.

### Characterization of large round multi-nucleated cells as potent progenitors of AD-CMG

**Figure 4:**
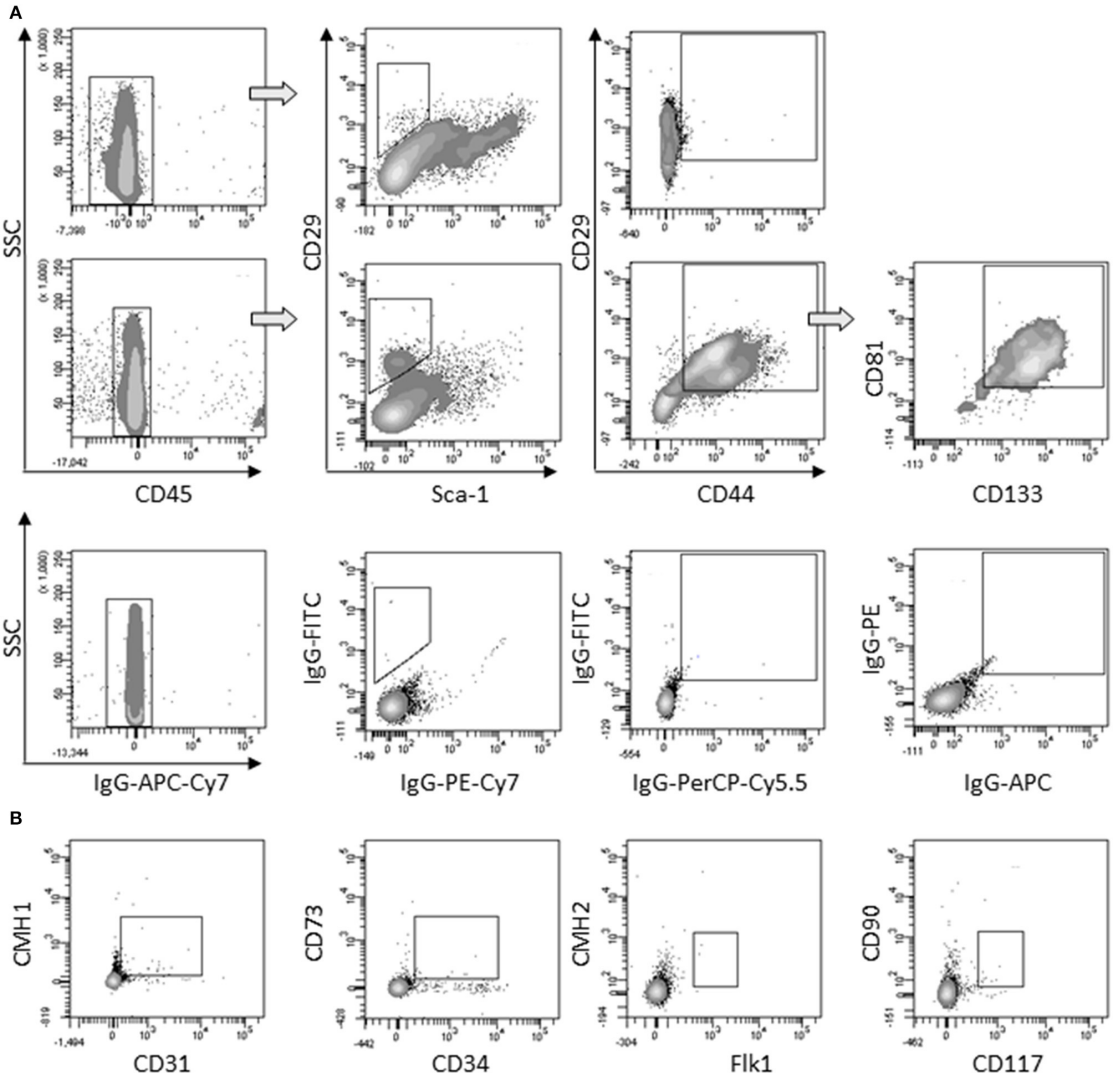
**Characterization of large round multi-nucleated cells (*c* cells). (A)** Flow cytometry analysis of surrounding cells (upper panels) and myogenic clusters (medium panels) picked off 8 days after plating. Cells were gated on CD45^−^ population and analyzed for CD29/Sca-1/CD44 expression. CD81/CD133 cells were gated on CD29^+^/CD44^+^ cells. Representative dot plots of specific (upper and medium panels) or isotype (lower panels) staining obtained in three separate experiments are shown. **(B)** Expression of CD31, CD34, CD73, CD90, CD117, CMH1, CMH2, and Flk-1 on CD45^−^ gated cells among picked myogenic clusters 8 days after plating. Abbreviation: SSC, side scatter.

First, we determined that the large round cells multi-nucleation resulted from cell fusion rather than endomitosis events (Figure S2). As large round multi-nucleated cells were mingled with other cells in cardiomiogenic clusters, they could hardly be set apart for phenotyping. To overcome this limit, individual clusters were selectively picked up among surrounding cells present in the culture plate at day 5, when large round multi-nucleated cells were numerous and predominant. The phenotype of selected cells was analyzed and compared to the phenotype of the remaining surrounding cells in order to bring out a specific phenotype that would be exclusively representative of myogenic clusters and in this case, mainly of large round multi-nucleated cells. Among CD45^−^ cells collected, a differential population of CD29^+^/Sca1^−^/CD44^+^ could be exclusively identified in the cells coming from the clusters, that represented 26,8 % (± 7.8) of the myogenic clusters cells and 1,7 % (± 0.3) of the environment cells in culture. This sub-population was positive for CD81 and CD133 (Figure 4A) and negative for CD31, CD34, CD73, CD90, CD117, CMH1, CMH2, and Flk1 (Figure 4B).

### AD-CMG progenitors require accessory cells to generate myogenic clusters

A cell population with the antigenic profile described above was identified in SVF, where it represented 0.1% (± 0.7) of total cells. To test whether this population has the potential to differentiate toward cardiomyocytes, we cultured a native cells subpopulation sorted by CD45^−^ CD44^+^, CD81^+^, or Sca1^−^; unfortunately, whatever the surface marker combination and the sorting technique (flow cytometry or magnetic cell sorting); sorted cells survived but no myogenic cluster emerged. Reconstitution of the crude SVF by mixing again cell populations after sorting also failed to induce contractile cluster formation in semi-solid medium, suggesting that the sorting process itself may impair cell properties. We hypothesized that cell separation before culture may have a negative impact on cardiogenic commitment by depleting specific surface proteins crucial for inducing proliferation and differentiation.

Alternatively to the sorting approach, clonal experiments were set up. Individual 5 days old clusters (when large round multi-nucleated cells were in majority) were picked up and seeded after limiting dilution to plate one cell in every 3 well. In these conditions, clonal cells survived but remained in a stationary state without proliferating and none of them gave rise to a myogenic cluster. Thus, AD-CMG could not be purified from crude SVF or myogenic clusters. However, these experiments revealed that AD-CMG progenitors required a specific cell environment to evolve into myogenic clusters.

**Figure 5:**
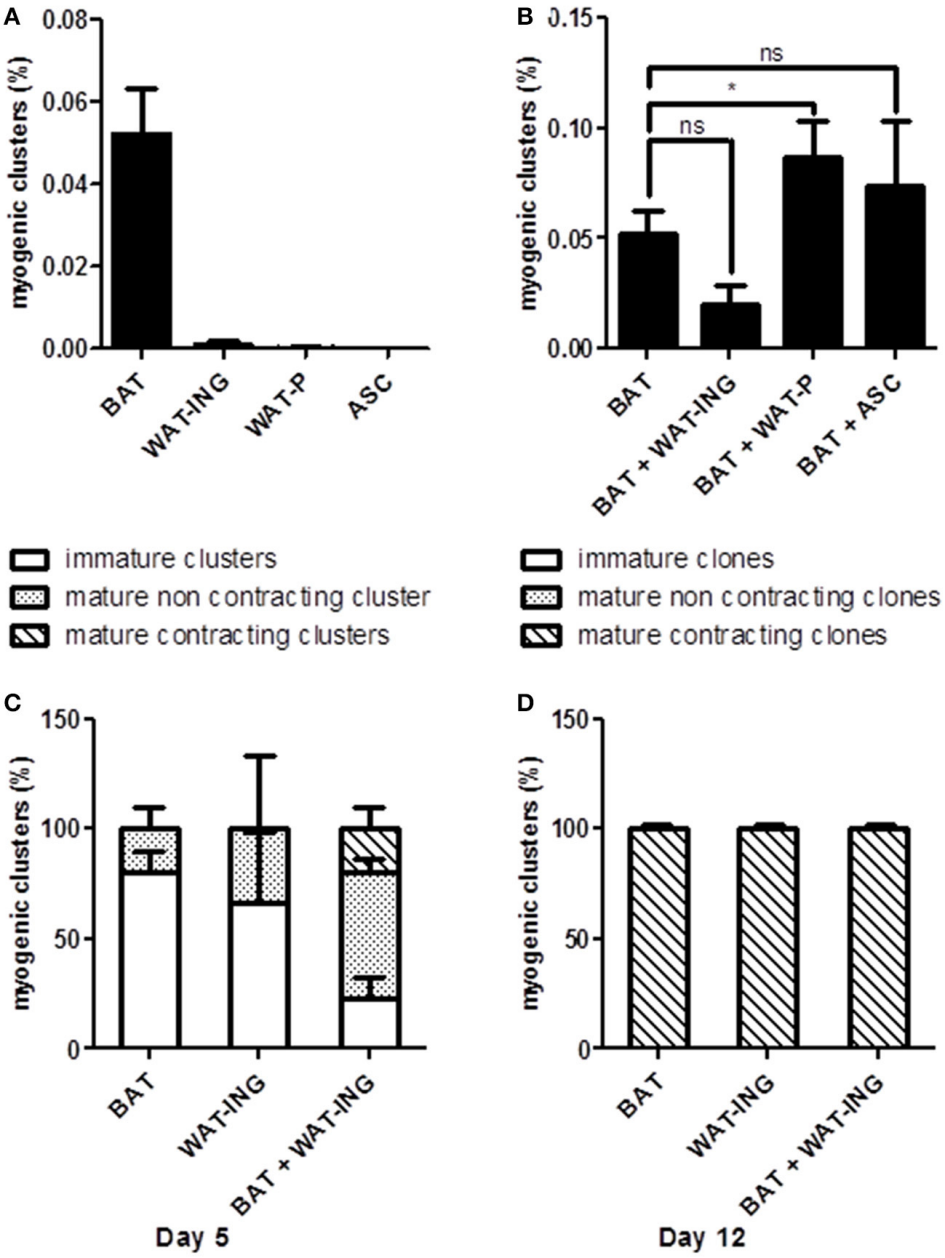
**Microenvironment influence on myogenic clusters emergence. (A)** Quantification of myogenic clusters generated 8 days after independent plating of BAT, WAT-ING, and WAT-P SVF cells and inguinal pad derived ASC. Results are expressed as percentage of total plated cells. **(B)** Quantification of myogenic clusters generated 8 days after co-culture of BAT SVF cells with WAT-ING or WAT-P SVF cells or inguinal pad derived ASC. Results are expressed as percentage of total plated BAT SVF cells. **(C,D)** Quantification of immature clusters, mature but not contractile clusters and mature and contractile clusters, 5 days **(C)** and 12 days **(D)** post plating. Results are expressed as percentage myogenic clusters. Abbreviations: BAT, brown adipose tissue; WAT-ING, inguinal white adipose tissue; WAT-P: peritoneal white adipose tissue. ^*^p < 0.05.

In order to better understand the role of microenvironment on AD-CMG emergence and to develop strategies to improve their yield, we investigated the influence of surrounding cells. To that purpose, adipose tissue derived SVF cells were cultured under our classical conditions in semi-solid medium to generate myogenic clusters and in some experiments, SVF cells from other adipose pads were added to provide different cellular environments. First, the intrinsic cardiomyogenic potential of SVF cells from brown (BAT), white inguinal (WAT-ING), and white peritoneal (WAT-P) adipose tissues as well as cultured ASC from inguinal fat pad was evaluated independently. It appeared that only SVF cells from BAT and WAT-ING could give rise to myogenic clusters (only one clone was detected in WAT-P among six experiments), with a better efficacy for BAT-derived SVF cells (Figure 5A); while cultured ASC did not generate any myogenic clusters by themselves. Then, we comparatively tested the addition of SVF cells from WAT-ING and WAT-P as well as ASC to BAT SVF cells. When co-cultured with BAT SVF cells, neither WAT-ING nor ASC did affect the potential of BAT SVF cells to generate myogenic clusters while WAT-P did potentiate the number of BAT SVF derived myogenic clusters (Figure 5B). Elsewhere, co-culture of BAT SVF cells with inguinal WAT-ING SVF induced striking acceleration in myogenic clusters maturation, characterized by beating events occurring as soon as 5 days after plating (Figure 5C), whereas equivalent beating activity was not observed before 12 days in control BAT SVF cultures (Figure 5D). This results suggested that some microenvironment were prone to support myogenic clusters emergence (i.e., SVF of WAT-P) while some other promote cardiogenic differentiation (i.e., SVF of WAT-ING).

## Discussion

We previously reported that cardiomyogenic cells (AD-CMG) could be obtained from white and brown adipose tissue *in vitro* and improved cardiac function in a myocardial infarction context (Planat-Benard et al., 2004; Leobon et al., 2009). Here, we reported complementary data on adipose-derived myogenic cluster formation and shed light on AD-CMG progenitors.

Kinetic analysis revealed morphological cell heterogeneity among clusters during their course of maturation. Molecular characterization confirmed that some sub-populations did exhibit cardiomyogenic phenotype and some of them also expressed markers of skeletal muscle, corresponding to an original mixed cardiac/skeletal muscle phenotype. Such a bi-phenotype has never been described before, excepted in the P19CL6 cell-line, a mouse embryonal carcinoma cell-line extensively used as an *in vitro* model of cardiomyocytes (Khodadadi et al., 2010).

In addition, cardiac phenotype appeared to be transient. We demonstrated that transfer of myogenic clusters into liquid medium allowed culture expansion. However the cardiac protein expression diminished with increasing passage numbers leading to a complete loss of their expression and beating activity after nine passages, while cells expressing skeletal transcript markers continued until passage 15. These findings suggest that AD-CMG phenotype was linked to specific culture conditions and was lost with time and expansion rate in liquid culture conditions.

In order to characterize AD-CMG progenitors, we focused on large round multi-nucleated cells strictly expressing marker of cardiomyocytes that emerged in semi-solid medium before myotube-like beating structures. We showed that multi-nucleation events in myogenic clusters resulted mostly from cell fusion events. Cell fusion is generally associated with skeletal muscle myoblasts forming myotubes but multinucleation in postnatal ventricular myocytes can occur by uncoupling karyokinesis from cytokinesis (Oh et al., 2003) and cardiomyocytes polyploidization was also described (Meckert et al., 2005; Liu et al., 2010), showing that multi-nucleation of AD-CMG (mature or not) is not incompatible with their cardiomyogeneity.

Distinct from hematopoietic stem cells (based on CD45+, CD117+) and endothelial progenitor cells (based on CD45−, CD34+, Flk−1+), large multi-nucleated AD-CMG progenitors displayed the following profile: CD45−/CD29+/Sca1−/CD44+/CD81+/CD133+/CD31−/CD34−/CD73−/CD90−/CD117−/Flk1−/CMH1−/CMH2−. They may be related to the muscle “Spoc” cells (Sca−1−,CD34−, CD45−, CD117−) (Winitsky et al., 2005), though the authors describe that no beating cells emerged when SVF cells were cultured under standard Spoc cell conditions. Moreover, AD-CMG progenitors differed from multipotential muscle cells (Sca−1+, CD34+) (Torrente et al., 2001) and resident cardiac stem cells (CD117+, Lin−) (Beltrami et al., 2003)/(CD117−, Sca1+) (Oh et al., 2003).

Previous studies could isolate an enriched cardiogenic progenitors population based on expression of CD133 in brown adipose tissue SVF among CD45^−^/ter119^−^/CD31^−^ cells (non hematopoietic, non endothelial cells) (Yamada et al., 2007). They differentiated into cardiomyocytes with a higher incidence than in other fractions and CD29^+^ (CD45^−^/ter119^−^/CD31^−^) cells, which had been previously described as a cardiomyogenic enriched population (Yamada et al., 2006). However, if sorted cells could differentiate into cardiomyocytes with a higher frequency than other fractions, the selected sub-population was not restricted to AD-CMG progenitors as >50% of them didn't generate cardiogenic cells.

A complete profile is still needed for purification but we showed that myogenic clusters emergence was largely impaired when AD-CMG progenitors were separated from supporting cells by cell sorting on the basis of markers such as CD44 CD81 or Sca1 or by clonal analysis, highlighting the important role of accessory cells for acquisition of cardiogenic phenotype. This requirement of sorted cells for accessory cells has been observed earlier: Zangiacomi et al showed that CD133^+^ cord blood stem/progenitor cells required co-culture with CD133^−^ cells to undergo neuronal differentiation (Zangiacomi et al., 2008).

This influence of surrounding cells on cardiogenic phenotype was underlined by our co-culture experiments showing that inguinal WAT SVF cells accelerated maturation of brown adipose tissue-derived cardiomyogenic cells. The fact that the emergence of myogenic phenotypes depends on time and culture conditions, raised some concerns about the role of the *in vitro* microenvironment regarding to secreted factors and cell contacts. According to our study, the myogenic potential of some adipose tissue cells requires surrounding cells and is silenced at the clonal level. The culture medium was optimized to contractile clusters formation however the subtle combination provided by the presence of cells couldn't be reproduced in the absence of supporting cells (data not shown). Thus, the initial step consisting in heterogenous SVF cells plating in semi-solid medium remains mandatory.

Requirement of surrounding cells evoked the stem cell “niche” concept described to condition and control stem cell fate. An abundant set of literature lead to the notion that adult stem cells are actually highly plastic and have the ability to cross lineage boundaries (Clarke and Frisen, 2001). It could be hypothesized that apart the well described mesenchymal progenitors (ASC), adult adipose tissue contained distinct highly plastic cells with skeletal/cardiac myogenic potentials. Considering that cardiac and skeletal muscle cells do not developmentally originate from a common progenitor, the obtained phenotypes could rather result from gain of potency through fusion events and/or differential distribution of potency in their progeny through asymmetric division. Indeed, multinucleated cells in adipose-derived myogenic clusters resulting from fusion events were observed (Figure S2). In addition, based on Numb repartition we highlighted asymmetric divisions as early events that occurred in small round mono-nucleated cells before emergence of other cell phenotypes (Figure S3). Numb is known to be asymmetrically distributed between mother and progeny cells in myogenic progenitors where it acts to block Notch-mediated repression of genes expressed in myogenic progenitor cells (Ruiz Gomez and Bate, 1997). In brown adipose tissue, asymmetric cell divisions may lead to a diversification of progenitors that would be subdivided to produce distinct populations of myogenic cells (likely cardiogenic and skeletal).

Much more beating cardiomyocytes are obtained from SVF of brown than inguinal adipose tissue. This can be linked to the vicinity of interscapular brown adipose tissue with muscle and to a common developmental origin. Indeed, Seale et al. described a common progenitor for brown fat and skeletal muscle (Seale et al., 2008). Thus, generation of cardiomyogenic cells from inguinal SVF is coherent with the emergence of ectopic brown adipocytes in such white adipose depots during cold acclimation in animals (Cousin et al., 1996) and it seems now very likely that, at least in some WAT pads, brown adipocytes can emerge from differentiation of brown preadipocytes or transdifferentiation of existing white adipocytes (for review, see Boss and Farmer, 2012).

Taken together, our data show that some non-adherent cells from adipose tissue SVF, when trapped in semi-solid culture medium undergo fusion and asymmetric division events leading to a cardiomyogenic phenotype. Cardiomyogenic phenotype can be influenced by accessory cells present in adipose tissue and may progressively be lost over cell expansion, suggesting that cardiogenic differentiation of such adipose-derived cells is merely acquired in culture and not a reflection of native biology.

### Conflict of interest statement

The authors declare that the research was conducted in the absence of any commercial or financial relationships that could be construed as a potential conflict of interest.

## Abbreviations

SVF: stromal vascular fraction
AD-CMG: adipose-derived cardiomyogenic cells
cTNT: cardiac troponin T
BAT: brown adipose tissue
WAT-ING: white inguinal adipose tissue
WAT-P: white peritoneal adipose tissue
ASC: adipose stromal cells
