# Supplementary material for: Mouse adipose tissue stromal cells give rise to skeletal and cardiomyogenic cell sub-populations
Source: Front Cell Dev Biol. 2014 Aug 25;2:42. doi: 10.3389/fcell.2014.00042 (PMC4206990; doi:10.3389/fcell.2014.00042)
Supplement: Supplementary file 1 [file Presentation1.ZIP › Video captions.pdf]

Video 1

The 1st video corresponds to a beating cardiomyogenic clone derived from adult mouse adipose tissue

Video 2

The 2nd video represents non beating cardiomyogenic cluster derived from adult mouse adipose tissue. The cluster cells are not beating as EDTA was added to the culture medium, showing that the cells that compose the cluster are cardiomyogenic cells that require extracellular Ca2+ for beating

Video 3

The 3rd video shows beating cluster cells after extracellular Ca2+ was re-added to the cuture medium, confirming requirement of the cells for extracellular calcium for their beating activity and so their cardiomyogenic phenotype rather than skeletal one.
