# Supplementary figures and images for: Mouse adipose tissue stromal cells give rise to skeletal and cardiomyogenic cell sub-populations
Source: Front Cell Dev Biol. 2014 Aug 25;2:42. doi: 10.3389/fcell.2014.00042 (PMC4206990; doi:10.3389/fcell.2014.00042)

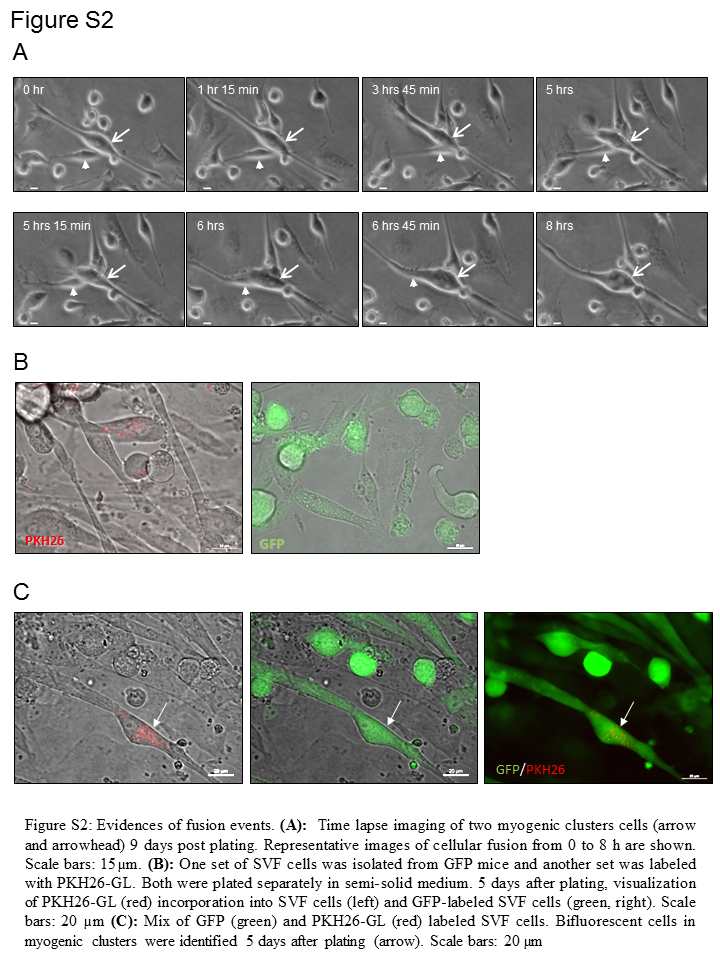

Supplement: Supplementary file 1 [file Presentation1.ZIP › Figure S2.TIF]

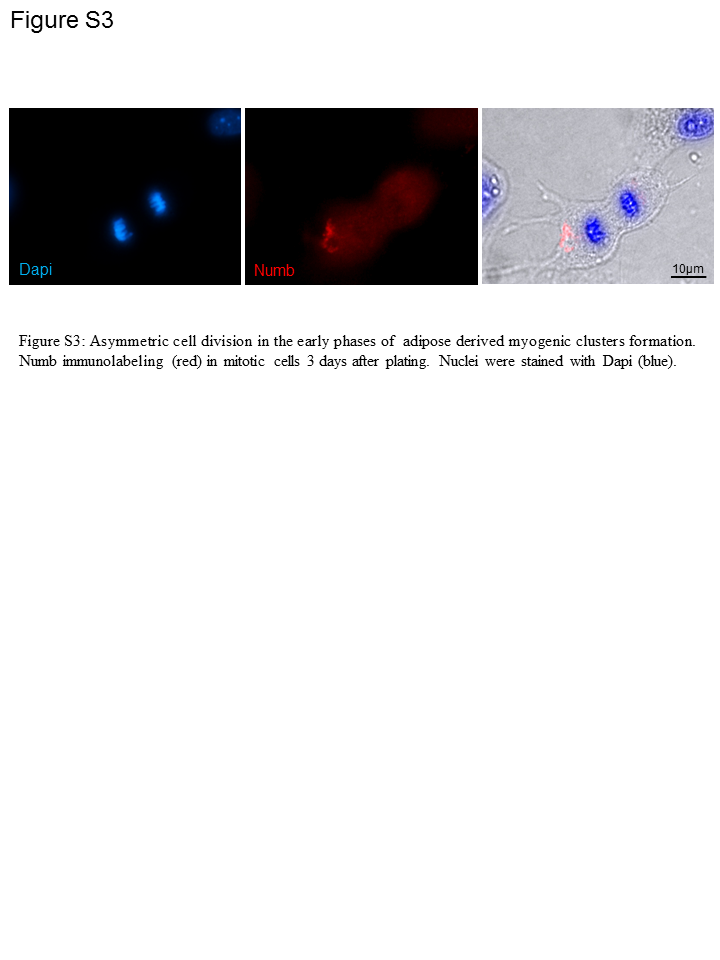

Supplement: Supplementary file 1 [file Presentation1.ZIP › Figure S3.TIF]

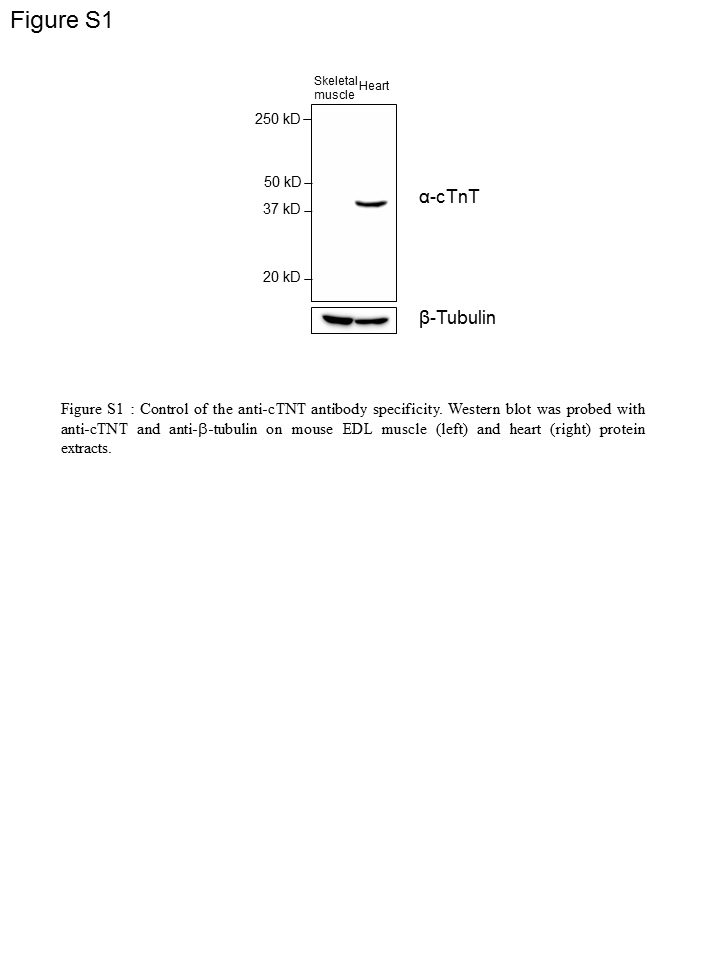

Supplement: Supplementary file 1 [file Presentation1.ZIP › Figure S1.TIF]
